# Dataset on a reliability generalization meta-analysis of the Oxford COVID-19 vaccine hesitancy scale

**DOI:** 10.1016/j.dib.2024.110451

**Published:** 2024-04-27

**Authors:** Kabiru Maitama Kura, Ramatu Abdulkareem Abubakar

**Affiliations:** a Faculty of Business and Logistics, Bahrain Polytechnic, Kingdom of Bahrain; b ABU Distance Learning Center, Ahmadu Bello University Zaria, Nigeria

**Keywords:** Heath data, Coronavirus, Public health, Data sharing, Raw data, Pandemic, Attitudes

## Abstract

The Oxford COVID-19 Vaccine Hesitancy Scale is a 7-item psychometric scale developed by Freeman and colleagues a year after detecting the first case of the disease in 2019. The scale assesses people's thoughts, feelings, and behavior toward COVID-19 vaccines. A comprehensive search of major electronic databases, including Scopus, Clarivate Analytics, and PubMed, was conducted to extract eligible articles for inclusion in this meta-analysis. This paper reports information on data collected for a reliability generalization meta-analysis of the Oxford COVID-19 Vaccine Hesitancy Scale. The dataset incorporates information on the average reliability of the scale as measured with Cronbach's alpha in 20 studies included in the meta-analysis. Several benefits can be derived from the dataset. In particular, the research community would find this dataset beneficial as it can enhance their understanding of the health challenges of COVID-19, helping them come up with better solutions to eradicate the disease.

Specifications Table

**Table:** 

Subject	Business, Management, and Decision Sciences.
Specific subject area	Public Health and Health Policy, Data Mining and Statistical Analysis
Data format	Aggregate, raw primary data (.csv), tables, and figures.
Type of data	Raw, coded data, screened, and partially analyzed.
Data collection	Data was collected from the published articles identified through a comprehensive search of electronic databases, including SCOPUS, Web of Science, Google Scholar, PsycINFO, JSTOR, and PubMed.
Data source location	Bahrain Polytechnic, Isa Town, Kingdom of Bahrain.
Data accessibility	Repository name: Mendeley Data Data identification number: 10.17632/cmp9p77nwc.4 Direct URL to data: https://data.mendeley.com/datasets/cmp9p77nwc/4

## 1 Value of the Data

• Researchers can use the dataset to replicate and evaluate the initial findings and explore new research questions related to the original meta-analysis, thereby contributing to the development of psychometric theory.
• Researchers and public health practitioners alike would find this dataset beneficial as it can enhance their understanding of the health challenges of COVID-19, helping them come up with better solutions to eradicate the disease.
• The dataset has the potential to trigger collaborations among researchers and professionals from diverse backgrounds, allowing them to initiate new and impactful research that could benefit society.
• Researchers can use the dataset cheaply and in transparent and credible ways as they do not need to repeat the entire process of generating the original data from the beginning.
• University and college teachers can adopt the dataset as a teaching resource, helping students learn quantitative research with original rather than synthetic data.

## 2 Background

Researchers have developed several measurement scales to understand better and address COVID-19 vaccine hesitancy [1,2]. Of these measurement scales, the Oxford COVID-19 Vaccine Hesitancy Scale [3] was the most widely cited by research communities [4,5]. The initial development and validation of [3] contribute to the theory and concept development in vaccine hesitancy research. Following the initial administration of the Oxford COVID-19 Vaccine Hesitancy Scale in the UK, several studies have utilized the scale to validate it across different research contexts worldwide. Given the abundance of published works that adopted/adapted the Oxford COVID-19 Vaccine Hesitancy Scale, it is imperative to integrate the findings of these studies by conducting a meta-analysis to get valuable insights into the scale's reliability across different research settings. Performing a meta-analysis would improve our understanding of the reliability and generalizability of the Oxford COVID-19 Vaccine Hesitancy Scale across various research settings. However, the success of every meta-analysis depends mainly on the availability of the data from published and unpublished works. Thus, the purpose of this paper was to provide detailed information on data collected for a reliability generalization meta-analysis of the Oxford COVID-19 Vaccine Hesitancy Scale.

## 3 Data Description

The data frame is structured into three columns and twelve rows ranging from study identification to publication status. Before conducting the analyses, some of the variables were coded. In particular, study design was coded with 1 = Experimental, 2 = Longitudinal, 3 = Cross-sectional, and 4 = Others. The publication status was coded into dummy variables with 1 = Published and 2 = Unpublished. The participants' gender in the included studies was coded with 1 = Male, 2 = Female, and 3 = Both. Finally, the study context was coded with nine dummy variables: 1 = United States, 2 =United Kingdom, 3 = Australia, 4 = Malaysia, 5 = Türkiye, 6 = Egypt, 7 = Taiwan, 8 = Croatia, and 9. = multi-country.

**Fig. 1:**
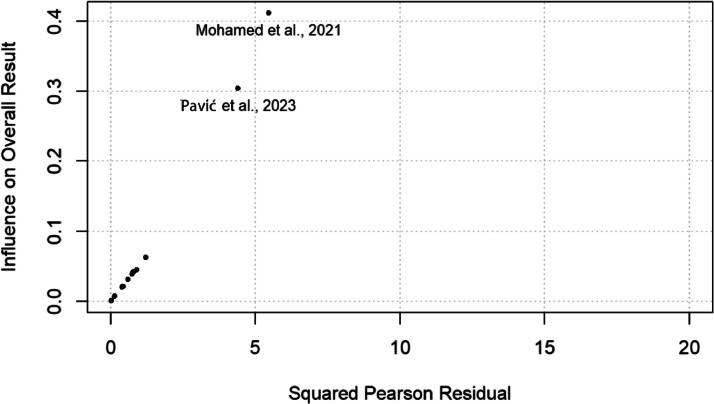
The Baujat plot. The figure provides information about how each individual study contributed to the observed heterogeneity in the meta-analysis. Each individual study included in the meta-analysis represents a point in the plot. Studies with the most significant variation from the overall effect size estimate and contributed to the observed heterogeneity are shown in the upper-left corner of the plot.

**Fig. 2:**
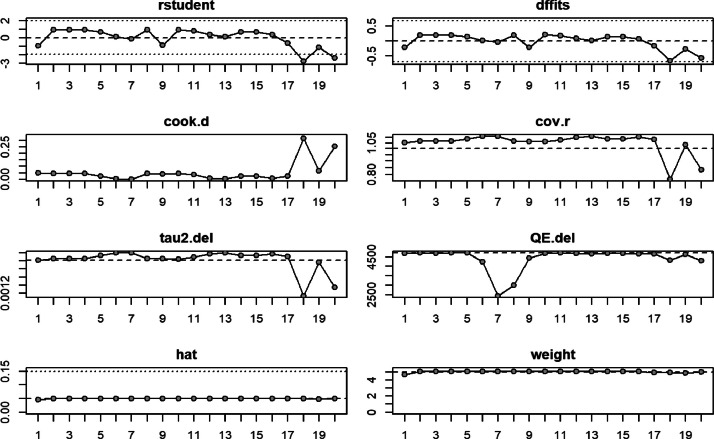
Outlier and influential study plots. The rstudent (standardised residuals), dffits (Difference in Fits), cook.d (Cook's distances), cov.r (covariance ratios), tau2.del (estimates of the amount of heterogeneity), QE.del (test statistics for heterogeneity), hat (Hat Values), and weight (Study Weights) were utilized to provide information about the diagnostic measures and influence of 20 studies examining the Reliability Generalization Meta-analysis of the Oxford COVID-19 Vaccine Hesitancy Scale.

**Fig. 3:**
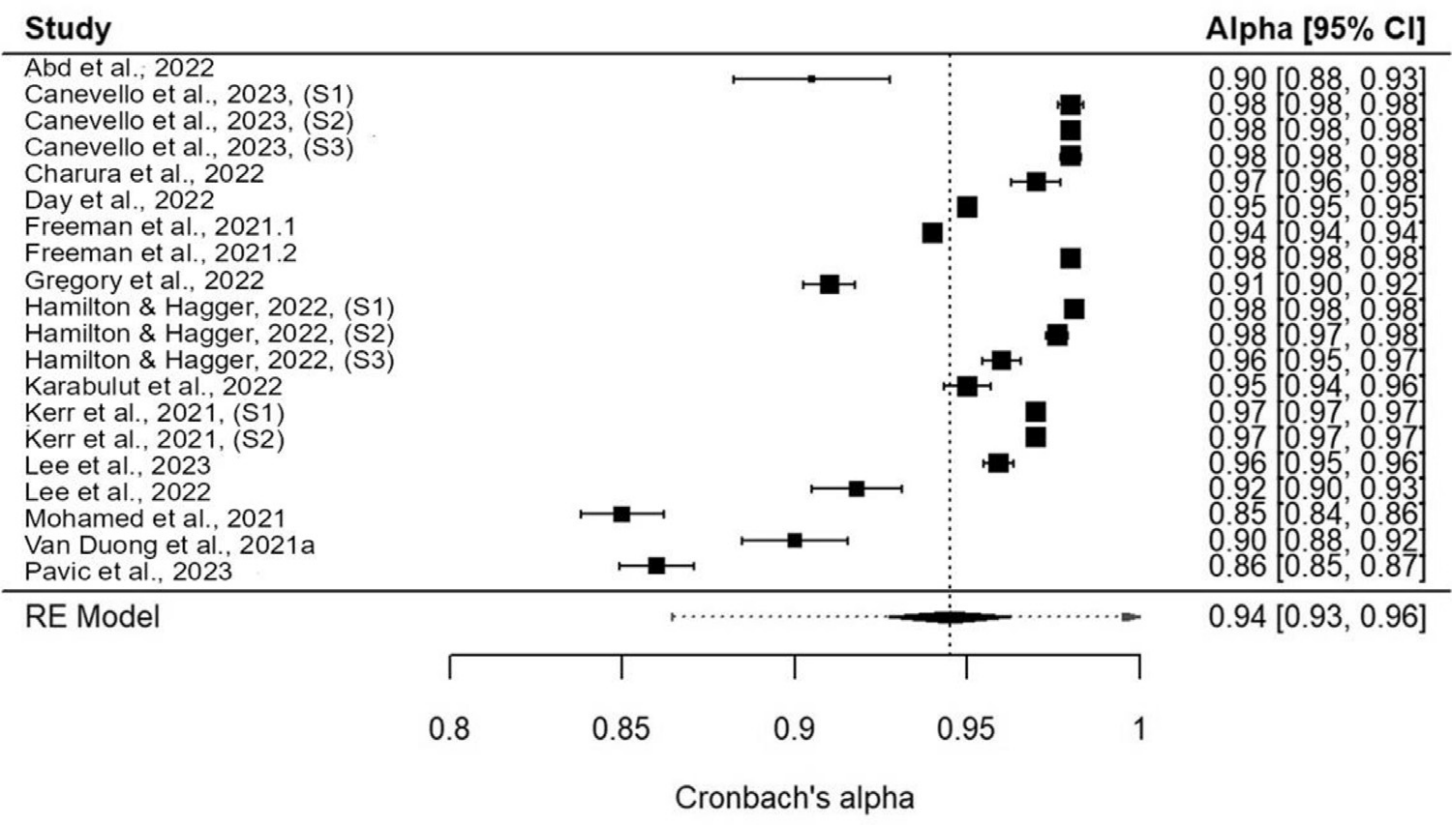
Forest plot. Forest plot displaying the alpha coefficients of individual studies with their corresponding 95 % confidence intervals. The pooled estimate of Cronbach's alpha coefficient across all studies is represented using a diamond at the bottom of the plot.

**Fig. 4:**
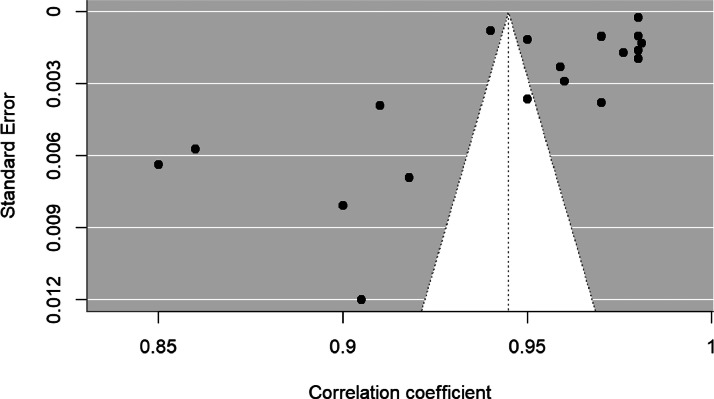
Depicts the funnel plot, providing information about the link between the standard error of the effect and effect sizes, plotted on the horizontal and vertical axes, respectively. Specifically, the Funnel plot suggests that as the standard error increases, the effect sizes vary across studies, with each dot representing an individual study included in the meta-analysis.

After creating the data frame, we organized the data into six tables and four figures. Table 1 presents the characteristics of the 20 studies included in the meta-analysis. Table 2 shows the Random-Effects Model. After presenting the heterogeneity statistics in Table 3, we show the Influence statistics in Table 4. The mixed-effects model with mean age, gender, study context, study quality, design of the study, and publication status as moderators is presented in Table 5. The assessment of publication bias is provided in Table 6. Fig. 1 depicts the Baujat plot to rule out studies that might contribute to heterogeneity. The graphical assessment outliers (i.e., influential studies) have been illustrated in Fig. 2. Finally, Fig. 3, Fig. 4 depict the forest and funnel plots, respectively.

**Table 1:** Studies characteristics.

Study ID	Authors	Sample size	Mean age	SD.	Gender	Reliability coefficient
1	Abd et al., 2022	159	25.6	3.7	Female	0.905
2	Canevello et al., 2023, (SI)	262	41.24	11.50	Both	0.98
3	Canevello et al., 2023, (S2)	1006	39.66	10.50	Both	0.98
4	Canevello et al., 2023, (S3)	393	39.04	12.40	Both	0.98
5	Charura et al., 2022	160	46.17	15.23	Both	0.97
6	Day et al., 2022	4683	60.6	13.3	Both	0.95
7	Freeman et al., 2021	15,014	47.2	17.5	Both	0.94
8	Freeman et al., 2021	16,445	43–2	18–1	Both	0.98
9	Gregory et al., 2022	2281	40.09	13.4	Both	0.91
10	Hamilton & Hagger, 2022, (SI)	522	47.40	14.83	Both	0.981
11	Hamilton & Hagger, 2022, (S2)	499	55.36	14.36	Both	0.976
12	Hamilton & Hagger, 2022, (S3)	479	52.14	14.55	Both	0.96
13	Karabulut et al., 2022	476	31.94	3.46	Both	0.95
14	Kerr et al., 2021, (SI)	2097	43.2	15.5	Both	0.97
15	Kerr et al., 2021, (S2)	2217	46.33	15.76	Both	0.97
16	Lee et al., 2023	798	30.46	12.68	Both	0.959
17	Lee et al., 2022	354	32.5	13.6	Female	0.918
18	Mohamed et al., 2021	1390	28.73	10.7	Both	0.85
19	Van Duong et al., 2021a	387	52.9	4.8	Both	0.90
20	Pavić et al., 2023	1500	42.6	13.1	Both	0.86

Study ID	Authors	Items used	Country	Study's quality	Study's design	Publication status

1	Abd et al., 2022	7	Egypt	3	Experimental	Published
2	Canevello et al., 2023, (SI)	7	United States	4	Cross-sectional	Published
3	Canevello et al., 2023, (S2)	7	United States	4	Cross-sectional	Published
4	Canevello et al., 2023, (S3)	7	United States	4	Cross-sectional	Published
5	Charura et al., 2022	7	Multi-country	3	Cross-sectional	Published
6	Day et al., 2022	7	Australia	3	Cross-sectional	Published
7	Freeman et al., 2021	7	United Kingdom	3	Cross-sectional	Published
8	Freeman et al., 2021	7	United Kingdom	4	Experimental	Published
9	Gregory et al., 2022	2	United States	2	Cross-sectional	Published
10	Hamilton & Hagger, 2022, (SI)	7	United States	4	Cross-sectional	Published
11	Hamilton & Hagger, 2022, (S2)	7	United States	4	Cross-sectional	Published
12	Hamilton & Hagger, 2022, (S3)	7	United States	4	Cross-sectional	Published
13	Karabulut et al., 2022	7	Türkiye	2	Cross-sectional	Published
14	Kerr et al., 2021, (SI)	7	United Kingdom	5	Experimental	Unpublished
15	Kerr et al., 2021, (S2)	7	United Kingdom	5	Experimental	Unpublished
16	Lee et al., 2023	7	Malaysia	2	Cross-sectional	Published
17	Lee et al., 2022	7	Malaysia	2	Cross-sectional	Published
18	Mohamed et al., 2021	7	Egypt	2	Cross-sectional	Published
19	Van Duong et al., 2021a	7	Taiwan	2	Cross-sectional	Published
20	Pavić et al., 2023	7	Croatia	2	Cross-sectional	Published

**Table 2:** Random-Effects Model (*k* = 20; tau^2 estimator: REML).

tau^2 (estimated amount of total heterogeneity)					0.0016 (SE = 0.0005)
tau (square root of estimated tau^2 value)					0.040
I^2 (total heterogeneity / total variability)					99.91 %
H^2 (total variability / sampling variability)					1125.570
Test for Heterogeneity:					
Q(df = 19) = 4720.788, p-val < 0.0001					
Model Results:					
estimate	se	zval	pval	ci.lb	ci.ub
0.9449	0.009	104.790	<0.0001	0.927	0.963***

Note: Signif. codes: 0 ‘***’ 0.001 ‘**’ 0.01 ‘*’ 0.05 ‘.’ 0.1′’ 1.

**Table 3:** Heterogeneity Statistics.

	0.002	0.001	0.004
tau^2	0.002	0.001	0.004
tau	0.040	0.031	0.059
I^2(%)	99.911	99.849	99.959
H^2	1125.573	660.021	2427.794

**Table 4:** Influence statistics.

	rstudent	dffits	cook.d	cov. r	tau2.del	QE.del	hat	weight	dfbs inf
Abd et al., 2022	−0.978	−0.216	0.047	1.050	0.002	4684.816	0.047	4.667	−0.216
Canevello et al., 2023,(SI)	0.892	0.206	0.043	1.066	0.002	4712.116	0.051	5.045	0.206
Canevello et al., 2023, (S2)	0.893	0.207	0.043	1.066	0.002	4686.218	0.051	5.054	0.207
Canevello et al., 2023, (S3)	0.893	0.206	0.043	1.066	0.002	4707.675	0.051	5.049	0.206
Charura et al., 2022	0.630	0.146	0.022	1.089	0.002	4719.293	0.050	5.015	0.146
Day et al., 2022	0.128	0.032	0.001	1.112	0.002	4225.563	0.051	5.053	0.032
Freeman et al., 2021	−0.121	−0.026	0.001	1.112	0.002	2441.039	0.051	5.055	−0.026
Freeman et al., 2021	0.893	0.207	0.043	1.066	0.002	2993.421	0.051	5.056	0.207
Gregory et al., 2022	−0.884	−0.203	0.042	1.064	0.002	4427.800	0.050	5.012	−0.203
Hamilton & Hagger, 2022, (SI)	0.919	0.213	0.046	1.063	0.002	4693.659	0.051	5.052	0.213
Hamilton & Hagger, 2022, (S2)	0.787	0.183	0.034	1.076	0.002	4719.888	0.051	5.048	0.183
Hamilton & Hagger, 2022,(S3)	0.377	0.089	0.008	1.104	0.002	4693.972	0.050	5.032	0.089
Karabulut et al., 2022	0.127	0.032	0.001	1.112	0.002	4672.054	0.050	5.018	0.032
Kerr et al., 2021, (Si)	0.632	0.147	0.022	1.089	0.002	4700.225	0.051	5.053	0.147
Kerr et al., 2021, (S2)	0.632	0.147	0.022	1.089	0.002	4698.996	0.051	5.054	0.147
Lee et al., 2023	0.352	0.083	0.007	1.106	0.002	4671.953	0.050	5.041	0.083
Lee et al., 2022	−0.669	−0.151	0.023	1.083	0.002	4649.229	0.049	4.920	−0.151
Mohamed et al., 2021	−2.803	−0.656	0.317	0.769	0.001	4310.891	0.049	4.940	−0.658
Van Duong et al., 2021a	−1.136	−0.258	0.065	1.034	0.002	4629.270	0.049	4.873	−0.258
Pavić et al. .,2023	−2.416	−0.565	0.254	0.835	0.001	4291.180	0.050	4.962	−0.566

**Table 5:** Mixed-effects model (*k* = 20; tau^2 estimator: REML).

tau^2 (estimated amount of residual heterogeneity)				0.000 (SE = 40.383)		
tau (square root of estimated tau^2 value):				0.000		
I^2 (residual heterogeneity / unaccounted variability)				0.00 %		
H^2 (unaccounted variability / sampling variability)				1.000		
R^2 (amount of heterogeneity accounted for)				0.00 %		
Test for Residual Heterogeneity:						
QE(df = 13) = 0.0870, p-val = 1.0000						
Model Results:						

	estimate	se	zval	pval	ci . 1 b	ci . ub

intrcpt	1.995	28.211	0.071	0.944	−53.298	57.287
meanage	−0.028	0.267	−0.105	0.916	−0.551	0.495
gender	−0.045	11.398	−0.004	0.997	−22.386	22.295
context	0.363	1.668	0.217	0.828	−2.907	3.632
quality	1.008	5.106	0.197	0.844	−9.000	11.015
design	0.678	6.594	0.103	0.918	−12.246	13.602
status	−0.023	16.205	−0.001	0.999	−31.785	31.738

**Table 6:** Assessment of publication bias.

Regression Test for Funnel Plot Asymmetry
Model: mixed-effects meta-regression model
Predictor: standard error
Test for Funnel Plot Asymmetry: *z* = −0.0851, *p* = 0.9321

## 4 Materials and Methods

A comprehensive search of electronic databases was performed to extract eligible studies for this meta-analysis, including SCOPUS, Web of Science, Google Scholar, PsycINFO, JSTOR, and PubMed. The following search terms were specifically applied: “Freeman COVID-19 vaccine hesitancy scale,” OR “COVID-19 vaccine hesitancy,” OR “vaccine hesitancy scale.” We specifically implemented a step-by-step procedure to select eligible articles for inclusion in this meta-analysis. In step one, we found 1839 records. These records were reduced to 1096 after removing duplicates in step two. In step three, 1032 records were excluded for several reasons, such as review articles (*n* = 17). Step three involves finding full-text records; our search yielded 64 full-text records in this step. However, of these records, the required statistics needed to extract data were missing from 44 articles, and emails were sent to authors to request the required information. After sending follow-up emails to the authors and receiving no response, these 44 records were excluded in the final step. Hence, 20 articles were included in the meta-analysis [6].

We utilized two open-source software to perform the meta-analysis, namely R (version 4.3.0) and the metafor package (version 4.0 [7,8]. Additionally, Cronbach's alpha was incorporated as the outcome measure. The result from the random-effects model shows that the model fits well with the data. We adopted the restricted maximum-likelihood estimator technique to establish the amount of heterogeneity (i.e., τ^2) [9]. Besides this, the Q-test for heterogeneity and the I2 statistic were also reported to understand further the heterogeneity among the studies included in the meta-analysis [10,11]. The result suggests that regardless of the results of the Q-test, heterogeneity has been detected among the studies included in the meta-analysis (τ^2 > 0) [12]. We employed Studentized residuals and Cook's distances to rule out the possibility of outliers or influence in the model context, as shown in Table 4 and Fig. 2 [8]. Statistically, studies that turn out to have a studentized residual larger than the 100 X (1 — 0.05/(2 x fc))th percentile of a standard normal distribution are deemed as potential outliers based on Bonferroni correction with two-tailed *a* = 0.05 for k studies included in the meta-analysis). Relatedly, we considered studies influential when a Cook's distance larger exceeds the median plus six times the interquartile range of the Cook's distances. We also utilized funnel plot, rank correlation test, as well as the regression test to determine the funnel plot asymmetry based on standard error for the observed outcomes as a predictor variable [13,14].

## Limitations

One of the main limitations of this data is its size. We were not fortunate enough to generate relatively more comprehensive data because many authors of the previous work did not respond to our emails requesting missing statistical information from their work despite several reminders sent to them. Given that recent studies [[15], [16], [17], [18]] suggest that COVID-19 vaccine hesitancy is still prevalent, it would be interesting to build on the current data by collecting more data to understand further the reliability of Oxford COVID-19 Vaccine Hesitancy scale and source of heterogeneity in the meta-analysis.

## Ethics Statement

This work does not require ethical approval since the data included in the meta-analysis was generated from published studies in which the authors have already obtained approval. Additionally, the work did not involve animals or human subjects.

## CRediT Author Statement

**Kabiru Maitama Kura:** Conceptualization, Methodology, Data screening, Reviewing and Editing for Quality, **Ramatu Abdulkareem Abubakar**: Analysis, Writing and drafting the manuscript.

## Data Availability

Reliability Generalization of the Oxford COVID-19 Vaccine Hesitancy Scale: Dataset and R-Script (Original data) (Mendeley Data)
